# The role of salivary lactoferrin as a potential biomarker for periodontal disease: a systematic review and meta-analysis

**DOI:** 10.3389/froh.2026.1812772

**Published:** 2026-05-22

**Authors:** Hoda Tayebi-Hillali, Yago Leira, João Botelho, Vanessa Machado, Carlota Rodrigues, Juan Blanco Carrión, Pedro Diz Dios

**Affiliations:** 1 Medical-Surgical Dentistry Research Group, Health Research Institute of Santiago de Compostela, School of Medicine and Dentistry, University of Santiago de Compostela, Santiago de Compostela, Spain; 2 Unit of Periodontology, School of Medicine and Dentistry, University of Santiago de Compostela, Santiago de Compostela, Spain; 3 NeuroAging Research Group, Clinical. Neurosciences Research Laboratories, Health Research Institute of Santiago de Compostela, University Clinical Hospital, Santiago de Compostela, Spain; 4 Egas Moniz Center for Interdisciplinary Research (CiiEM), Egas Moniz School of Health & Science, Almada, Portugal

**Keywords:** biomarker, gingival crevicular fluid, lactoferrin, periodontal diagnosis, periodontal disease, periodontal monitoring, saliva

## Abstract

**Aim:**

To systematically review and meta-analyse the diagnostic potential of salivary lactoferrin for periodontal disease monitoring.

**Materials and methods:**

A comprehensive electronic search was conducted across five databases without time restrictions, up to January 2026. Studies quantifying lactoferrin in saliva or gingival crevicular fluid in individuals with clinically diagnosed periodontal disease were included. Eligible designs were case-control and longitudinal pre-post intervention studies. Risk of bias was assessed via the Newcastle-Ottawa Scale for case control studies and ROBINS-I tool for intervention studies, and evidence certainty was graded using GRADE. Meta-analysis with stratified subgroup analyses was performed to explore inter-study variability.

**Results:**

Nineteen studies were included and 11 contributed to the meta-analysis. Compared to periodontally healthy subjects, lactoferrin levels were significantly higher in individuals with periodontal disease: Ratio of Means (ROM) = 1.77 (95% CI: 1.44–2.24) in unstimulated saliva, ROM = 2.14 (95% CI: 1.38–3.32) in stimulated saliva, and ROM = 8.63 (95% CI: 2.68–27.87) in gingival crevicular fluid. Lactoferrin levels decreased after mechanical periodontal treatment, notably in gingival crevicular fluid. Considerable methodological heterogeneity and variable study quality were noted. Meta-analysis of intervention studies was not feasible due to inconsistent protocols and reporting.

**Conclusion:**

Elevated lactoferrin levels in periodontitis and their reduction post-treatment highlights its potential as a biomarker for periodontal disease monitoring. However, methodological standardization and robust studies are needed to confirm clinical applicability.

**Systematic Review Registration:**

identifier: CRD420251017848.

## 1 Background

Periodontal disease diagnosis currently relies on clinical parameters that primarily reflect past tissue destruction, limiting their ability to detect early-stage disease and differentiate between active and inactive lesions. These limitations, coupled with the subjective nature of clinical evaluations, underscore the need for objective and sensitive diagnostic tools (1–5). In this context, salivary biomarkers have emerged as promising candidates for early detection and monitoring of periodontal disease progression (6, 7).

Multiple salivary biomarkers, including pro-inflammatory cytokines (e.g., IL-1β, IL-6), chemokines (e.g., MCP-1), and growth factors (e.g., VEGF, HGF), have demonstrated elevated levels in individuals with periodontitis (8–19), while paradoxically other biomarkers such as IL-10, IL-17, MMP-8, and TNF-α may be reduced (20–23). Recent advances in proteomic and metabolomic profiling have led to the discovery of novel biomarkers (e.g., CD9, CD81, suPAR, galectin-1, S100A8, LDH, AST) with utility in disease diagnosis, activity assessment, and therapeutic monitoring (21, 24–26).

Lactoferrin (LF) stands out as a particularly promising salivary biomarker. LF is a multifunctional iron-binding glycoprotein of the transferrin family, first identified in bovine milk (1939) and later in human milk (1960) and is abundantly stored in neutrophil granules (27, 28). Beyond iron metabolism, LF plays roles in modulating immune responses, controlling cell proliferation, and exerting antimicrobial, antiviral, and antiparasitic effects (29, 30). Importantly, as LF is abundant in the specific granules of neutrophils (31), its levels in saliva and GCF directly reflect the recruitment and activation of these key inflammatory cells within the diseased periodontium. Accordingly, elevated LF concentrations are expected in affected patients, serving as a direct proxy for the local neutrophil activity and the magnitude of the host inflammatory response (51).

At the molecular level, LF exists in two principal bioactive conformations: the iron-depleted apo-LF (<5% iron saturation) and the iron-saturated holo-LF (>85% iron saturation). These isoforms exhibit distinct functional profiles, with apo-LF predominantly associated with immunomodulatory and antimicrobial activities, whereas holo-LF has been implicated in differential signalling pathways, including those potentially linked to tumorigenesis (32–36).

LF exhibits species- and strain-specific antimicrobial effects against periodontal pathogens. In *Aggregatibacter actinomycetemcomitans* (Aa), a key agent in aggressive periodontitis, apo-LF shows limited activity against clinical isolates, though laboratory strains are more susceptible at acidic pH. Holo-LF inhibits Aa adhesion to epithelial cells, an effect not shared by apo-LF or iron alone (37, 38). In *Porphyromonas gingivalis* (Pg), a keystone pathogen in chronic periodontitis (CP), LF exerts bacteriostatic effects under iron-limited conditions via haemoglobin receptor interaction, though this effect is abolished by the presence of hemin. Moreover, LF degradation by Pg proteases compromises its antimicrobial efficacy (39). LF also binds to and inhibits epithelial adhesion of *Prevotella intermedia* and *P. nigrescens*, though with lower affinity than to Pg, highlighting LF's role in modulating early microbial colonization (40, 41).

Owing to its functional versatility, its presence in both saliva and gingival crevicular fluid (GCF), and its ability to interact with key oral pathogens, LF has emerged as a promising candidate biomarker for periodontal disease. Its investigation may enhance our understanding of the underlying pathophysiological mechanisms of periodontitis and support the development of more objective and sensitive diagnostic approaches.

Therefore, the present study aimed to systematically review and meta-analyse the available evidence on salivary LF as a potential biomarker for periodontal disease. Specifically, the objectives were: 1. To compare LF levels in saliva and GCF between individuals with periodontal disease and those with a clinically healthy periodontium; and 2. To evaluate longitudinal changes in salivary LF concentrations in response to periodontal treatment.

## 2 Matherial and methods

### 2.1 PROSPERO registration

The protocol for this systematic review and meta-analysis was registered in the PROSPERO database under the reference code CRD420251017848. The protocol followed the instructions provided in the Cochrane Handbook for Systematic Reviews of Interventions—Second Edition (42), and the results will be presented in accordance with the Preferred Reporting Items for Systematic Reviews and Meta-Analyses (PRISMA) guidelines (43).

### 2.2 PECO and PICO questions

This review was developed to answer the following focused questions:

PECO Question 1: “In individuals with periodontitis (Participants), does periodontitis (Exposure) influence lactoferrin (LF) levels in saliva and/or gingival crevicular fluid (GCF) (Outcome), and can these LF levels be used as a potential biomarker of disease presence or activity?”.
PICO Question 2: “In individuals with periodontitis (Participants), does mechanical periodontal treatment (Intervention) reduce LF levels in saliva and/or GCF compared to LF levels in saliva and/or GCF before periodontal treatment (Comparison), and can these levels serve as a potential indicator of favourable therapeutic response (Outcome)?”.

### 2.3 Data sources

Relevant studies on this topic were identified through a targeted search conducted in PubMed, Scopus, Web of Science, Embase, and Cochrane Library databases, up to January 2026. The following keywords, along with synonyms and free-text terms, were used in all databases to maximize search sensitivity: [(lactoferrin) OR (lactotransferrin)] AND [(periodontal disease) OR (periodontitis)]. The detailed search strategy for each database is provided in Supplementary Table S1.

### 2.4 Eligibility criteria and study selection process

The following inclusion criteria were applied for study selection in this review: case-control studies measuring salivary LF concentrations in humans with periodontal disease and healthy controls; and studies evaluating LF levels in individuals with periodontal disease before and after mechanical periodontal treatment, without restrictions on publication date. The exclusion criteria included: studies conducted in animal models; studies involving individuals diagnosed with gingivitis or considered periodontally healthy; retracted articles, letters, commentaries, editorials, opinion pieces, *in vitro* studies, and review articles.

The records retrieved after merging results from the databases were independently screened by two investigators (HT and PD) using EndNote 21 software (Clarivate Analytics, Philadelphia, Pennsylvania, USA). Initially, titles and abstracts were reviewed to identify candidate articles for full-text eligibility assessment. During this process, inter-reviewer agreement was quantified using Cohen's kappa coefficient (*κ*). Discrepancies were discussed and resolved with the involvement of a third investigator (YL). Finally, the definitive selection of studies was conducted based on the pre-established inclusion and exclusion criteria (JB and VM).

### 2.5 Data extraction variables

Two investigators (HT and PD) independently extracted data using a standardized form for each eligible study. Extracted variables included: first author and year of publication, country where the study was conducted, number of participants, sex and age distribution, type of periodontal disease, number and type of samples collected (unstimulated saliva, stimulated saliva, or GCF), baseline LF levels in case-control studies, and LF levels before and after mechanical treatment in intervention studies.

### 2.6 Quantitative synthesis

For the meta-analysis, continuous data were expressed as means and standard deviations (SD) and analysed using the ratio of means (ROM) along with corresponding 95% confidence intervals (CI) (42, 44, 45). The analysis was stratified by type of periodontitis, and for each subgroup (unstimulated saliva, stimulated saliva, or GCF), the DerSimonian–Laird random-effects model was applied, with estimations performed using R software version 3.4.1 (46). All random-effects meta-analyses and forest plot constructions were conducted using the “meta” package (47). Statistical heterogeneity among studies was evaluated using the *I*^2^ statistic and Cochran's *Q* test (significance threshold *p* < 0.1), while overall homogeneity was assessed via the *χ*^2^ test. Heterogeneity was classified as low (*I*^2^ < 50%) or high (*I*^2^ > 50%). All tests were two-tailed with an alpha level set at 0.05. Publication bias assessment was planned but not feasible due to the limited number of studies included per estimate (fewer than 10). Overall effect estimates are presented with corresponding 95% Cis (42).

### 2.7 Risk of bias assessment and certainty of evidence

To minimize the risk of bias, 2 reviewers (CR and JB) independently assessed the included studies using the Newcastle-Ottawa Scale (NOS) for case-control and the ROBINS-I tool for non-randomized studies-of interventions (48, 49). For the NOS, the assessment employed a star-rating system across 3 domains: selection, comparability, and outcome/exposure assessment. Studies scoring 8–9 stars were deemed “high quality,” those scoring 7 stars as “moderate quality,” and those with 6 or fewer stars as “low quality.” For the ROBINS-I tool, low risk of bias was indicated in green, moderate risk in orange, serious risk in red, and critical risk in black for each domain.

The overall quality and certainty of the evidence regarding salivary LF levels as a biomarker for periodontal disease and in response to mechanical treatment were appraised using the Grading of Recommendations Assessment, Development and Evaluation framework (GRADE) (50). Two reviewers (HT and CR) independently assessed the quality of evidence and robustness of recommendations, considering the following criteria: risk of bias within individual studies, consistency and precision of effect estimates, directness of evidence applicability, and potential publication bias. Discrepancies were resolved through discussion with a third reviewer (PD).

## 3 Results

**Figure 1:**
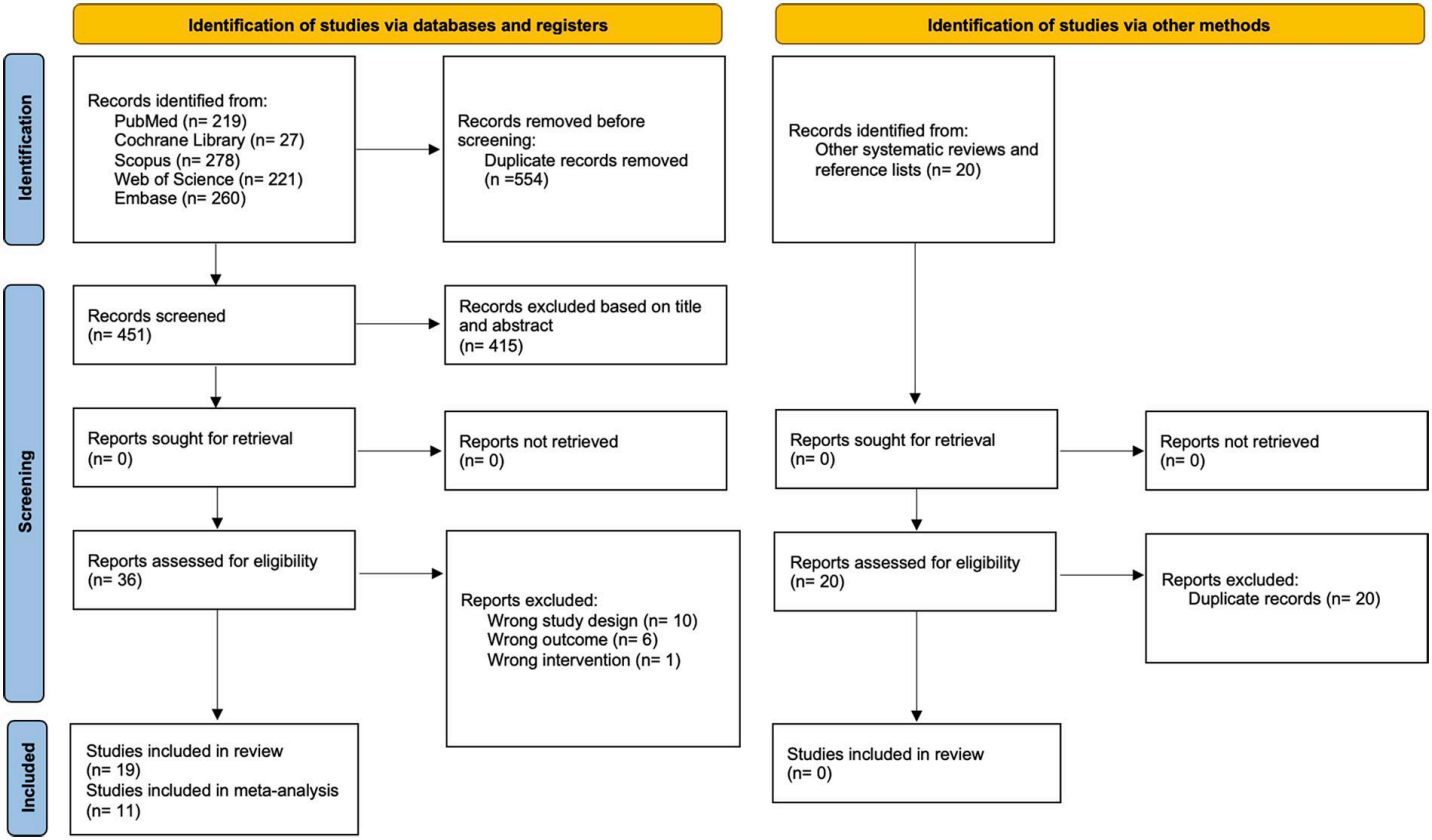
PRISMA flow diagram.

A total of 1,005 potentially eligible articles were identified through systematic searches across bibliographic databases. After removal of duplicates and application of the predefined eligibility criteria, 19 studies met the inclusion criteria. Of these, 11 provided sufficient quantitative data to be included in the meta-analysis (Figure 1). The excluded articles and reasons for exclusion are provided in Supplementary Table S2.

The included articles spanned a publication period from 1983 to 2025. The overall inter-reviewer agreement was 90.68%, with a Cohen's kappa coefficient of 0.90. Among the selected observational studies, case-control designs predominated (*n* = 16); in addition, 6 non-randomized clinical trials employing a pre-post intervention design were included. Notably, 3 of the 6 intervention studies were also classified within the case-control group, as they assessed baseline LF levels in both periodontitis patients and periodontally healthy individuals (51–53).

### 3.1 Observational studies

LF concentrations in saliva and GCF have been evaluated as potential diagnostic biomarkers of periodontal disease, through comparative analyses between affected individuals and periodontally healthy controls.

#### 3.1.1 Study characteristics

The included studies represented a broad geographical distribution, with a predominance of research conducted in Europe (51, 54–57) and Asia (52, 53, 58–62), as well as contributions from other countries including the United States (38, 63), Brazil (64), and Nigeria (65). A detailed overview of the demographic and methodological features of the included studies is presented in Tables 1–3.

**Table 1:** Main characteristics and findings of studies published on lactoferrin levels in unstimulated saliva samples from individuals with periodontal disease.

Author and year	Country	Periodontal diagnosis N (male/female)	Age mean ± SD (range)	Exclusion criteria	Baseline lf levels in cases and controls mean ± SD (range)	*p*-value
Wu et al. (60)	China	GAgP: 10 (5m/5f)	GAgP: 24.8 ± 3.8y(NA)	Smokers	GAgP: 6.8 ± 0.8 µg/mL	<0.001
				Alcohol consumers		
		Controls: 10 (5m/5f)	Controls: 24 ± 0.71y(NA)	Systemic diseases	Controls: 8.9 ± 0.5 µg/mL	
				Pregnancy/Nursing		
Lourenço et al. (64)	Brazil	CP: 16 (9m/7f)	CP: 38 ± 9y(NA)	Radiotherapy and/or chemotherapy	CP: 32.7 ± 12.9 µg/mL	<0.0001
		Controls: 22 (10m/12f)	Controls: 33 ± 10y(NA)	Antibiotics	Controls: 12.7 ± 8.0 µg/mL	
				Anti-inflammatory drugs(a)(c)		
Fine et al. (94)	USA	CP(Aa+): 13(NA)	Total: 14.96 ± 3.3y(NA)	Systemic diseases	CP(Aa+): NA	≤0.05
		Controls (Aa+): 7(NA)			Controls (Aa+): 8.62 ± 7.76 µg/mL	
					Controls (Aa-): 21.21 ± 1.87 µg/mL	
		Controls (Aa-): 10(NA)				
		Total: 30 (12m/18f)				
Wu et al. (61)	Taiwan	L/GSP: 30 (13m/17f)	L/GSP: 44.1 ± 12.0y(NA)	Smokers	L/GSP: 15.737 ± 7.826 µg/mL^†^	NS
				Systemic diseases		
		Controls: 27 (13m/14f)	Controls: 40.6 ± 16.5y(NA)	Pregnancy/Nursing	Controls: 10.725 ± 8.49 µg/mL^†^	
				Medication(b)		
Lee et al. (58)	Taiwan	SCP: 34 (16m/18f)	Total: NA(25–75y)	Smokers	SCP: 15.801 ± 4.43 µg/mL^†^	0.02
		Controls: 20 (10m/10f)		Systemic diseases	Controls: 10.877 ± 11.21 µg/mL^†^	
Ramenzoni et al. (56)	Germany	GCP III-B: 10 (6m/4f)	GCP III-B: NE (30–69y)	Smokers (>10 cig/day)	GCP III-B: NA	NS
				Systemic diseases		
		Controls: 10 (2m/8f)	Controls: NE (17–59y)	Pregnancy/ Nursing	Controls: NA	0.001
Ramenzoni et al. (57)	Germany	GCPIII-B: 20 (15m/5f)	GCPIII-B: 51.1 ± 12.3y(NA)	(a)	GCPIII-B: 15.9 ± 1.7 µg/mL	
		Controls: 20 (9m/11f)	Controls: 50.4 ± 11.2y(NA)		Controls: 9.3 ± 1.9 µg/mL	

N, number of participants; SD, standard deviation; m/f, male/female; GAgP, generalized aggressive periodontitis; GCP, generalized chronic periodontitis; CP, chronic periodontitis; CP (Aa+), chronic periodontitis with *Aggregatibacter actinomycetemcomitans*; CP (Aa-), chronic periodontitis without *Aggregatibacter actinomycetemcomitans*; LSP, localized severe periodontitis; GSP, generalized severe periodontitis; SCP, severe chronic periodontitis; GCP III-B, generalized chronic periodontitis stage III grade B; NA, not available; y, years; cig., cigarette; (a), smokers were included; (b), anti-inflammatory drugs, steroids, bisphosphonates, or antibiotics, for more than 2 weeks; (c) only HIV-negative groups were analyzed; LF, lactoferrin; NS, not significant.

† Calculated based on the median and interquartile range.

**Table 2:** Main characteristics and findings of studies on lactoferrin levels in stimulated saliva samples from individuals with periodontal disease.

Author and year	Country	Periodontal diagnosis N (male/female)	Age mean ± SD (range)	Exclusion criteria	Baseline lf levels in cases and controls mean ± SD (range)	*p*-value
Suomalainen et al. (51)	Finland	LJP: 7 (1m/6f) Controls: 7 (1m/6f)	LJP: 17.5y(14–20y) Controls: 23y(20–26y)	Smokers	NA	NS
Groenink et al. (54)	Netherlands	AgP(Aa+): 20 (7m/13f) Controls: 19 (1m/18f)	AgP(Aa+): 42y(17–57y) Controls: 23y(19–35y)	(a)	AgP(Aa+): 12.0 ± 10.5 µg/mL Controls: 5.0 ± 2.3 µg/mL	0.01
Glimvall et al. (55)	Switzerland	CP: 17 (11m/6f) Controls: 17 (5m/12f)	CP: 58 ± 11y(NA) Controls: 39 ± 14y(NA)	(b)	CP: 2.71 ± 2.36 µg/mL Controls: 1.12 ± 1.98 µg/mL	0.05
Ramenzoni et al. (56)	Germany	GCP III-B: 10 (6m/4f) Controls: 10 (2m/8f)	GCP III-B: NE (30–69y) Controls: NE (17–59y)	Smokers (>10 cig/day) Systemic diseases Pregnancy/ Nursing	GCP III-B: NA Controls: NA	NS
Ramenzoni et al. (57)	Germany	GCPIII-B: 20 (15m/5f) Controls: 20 (9m/11f)	GCPIII-B: 51.1 ± 12.3y(NA) Controls: 50.4 ± 11.2y(NA)	(c)	GCPIII-B: 28.5 ± 3.5 µg/mL Controls: 9.6 ± 1.8 µg/mL	0.001
Orhue et al. (65)	Nigeria	CP: 51 (23m/28f) Controls: 51 (24m/27f)	CP: NA (> 41y) Controls: NA(<41y)	Smokers Pregnancy Menopause/Postmenopause Inflammatory disorders	CP: 6.74 ± 4.35 µg/mL† Controls: 5.27 ± 4.21 µg/mL^†^	0.001
Arab et al. (62)	Saudi Arabia	LCP: 89(NA) GCP: 49(NA) Controls: 303(NA) Total: 441 (213m/228f)	LCP: 59.8 ± 11.4y(NA) GCP: 64.3 ± 11.8y(NA) Controls: 42.6 ± 15.5y(NA)	(d)	LCP: 2,180 ± 2,910 µg/mL GCP: 1,680 ± 1,100 µg/mL Controls: 1,680 ± 1,380 µg/mL	NS

N, number of participants; SD, standard deviation; m/f, male/female; LJP, localized juvenile periodontitis; AgP (Aa+), aggressive periodontitis with *Aggregatibacter actinomycetemcomitans*; CP, chronic periodontitis; GCP III-B, generalized chronic periodontitis stage III grade B; LCP, localized chronic periodontitis; GCP, generalized chronic periodontitis; NA, not available; y, years; cig., cigarette; (a) smokers, systemic diseases, and medication were included; (b), Smokers and diabetic individuals were included; (c) smokers were included; (d) smokers, diabetic individuals, and individuals with cardiovascular disease were included; LF, lactoferrin; NS, not significant.

† The standard deviation was calculated based on the standard error of the mean provided in the paper.

**Table 3:** Main characteristics and results of published studies on lactoferrin levels in crevicular gingival fluid samples from individuals with periodontal diseases.

Author and year	Country	Periodontal diagnosis N (male/female)	Age mean ± SD (range)	Exclusion criteria	Baseline lf levels in cases and controls mean ± SD (range)	*p*-value
Friedman et al. (63)	USA	CP: 17(NA) LJP: 12(NA) Controls: 7(NA) Total: 36(NA)	CP: NA(27–60y) LJP: NA(12–22y) Controls: NA(20–25y)	Smokers Systemic diseases	CP: 1,400 ± 790 µg/mL LJP: 1,700 ± 530 µg/mL Controls: 630 ± 320 µg/mL	<0.01
Suomalainen et al. (51)	Finland	LJP: 7 (1m/6f) Controls: 7 (1m/6f)	LJP: 17.5y(14–20y) Controls: 23y(20–26y)	Smokers	NA	NS
Tsai et al. (58)	Taiwan	CP: 13 (8m/5f) Controls: 5 (3m/2f)	CP: NA(26–50y) Controls: NA(24–27y)	Systemic diseases Medication	CP: 0.552 ± 0.419 µg/mL Controls: 0.069 ± 0.022 µg/mL	0.001
Wei et al. (59)	Taiwan	CP: 19 (11m/8f) Controls: 8 (3m/5f)	CP: 45.1 ± 10.0y(NA) Controls: 26.6 ± 1.9y(NA)	Smokers Systemic diseases	CP: 192.75 ± 156.54 µg/mL Controls: 193 ± 168.12 µg/mL	NS
Yadav et al. (52)	India	GCP: 25(NA) Controls: 25(NA) Total: 50 (20m/30f)	Total: NA(25–54y)	Smokers Systemic diseases Pregnancy/Nursing	GCP: 1.86 ± 0.091 µg/mL Controls: 0.075 ± 0.007 µg/mL	0.001
Ramenzoni et al. (56)	Germany	GCPIII-B: 10 (6m/4f) Controls: 10 (2m/8f)	GCP: NA(30–69y) Controls: NA(17–59y)	Smokers (>10 cig/day) Systemic diseases Pregnancy/Nursing	GCP: 1.656 ± 0.139 µg/mL Controls: 0.508 ± 0.002 µg/mL	<0.05

N, number of participants; SD, standard deviation; m/f, male/female; CP, chronic periodontitis; LJP, localized juvenile periodontitis; GCP, generalized chronic periodontitis; III-B, stage III grade B; NA, not available; y, years; cig., cigarette; LF, Lactoferrin; NS, not significant.

Sex distribution was balanced across the studies (male-to-female ratio = 0.915). The mean age among cases was 45.1 ± 10.0 years, whereas among controls it was 26.6 ± 1.9 years. CP was the most frequently reported type, accounting for 82.3% of cases included in case-control studies (373 CP cases vs. 79 cases with aggressive periodontitis [AgP] or juvenile localized periodontitis [JLP]). Most studies excluded participants who were smokers, pregnant or lactating women, and individuals with systemic diseases such as diabetes mellitus.

In terms of sample collection, unstimulated saliva was obtained in 7 studies, stimulated saliva in another 7, and GCF in 6 studies. For LF quantification, the enzyme-linked immunosorbent assay (ELISA) was the most employed method, used in 14 of the 16 included studies (87.5%). The remaining studies utilized electroimmunodiffusion and immunoenzymometric assay (IEMA), respectively (51, 63).

#### 3.1.2 Risk of bias assessment

Among the case-control studies, three were rated as high quality, achieving a score of 8 or 9 on the NOS (53, 56, 62). These studies demonstrated methodological rigor in participant selection, group comparability, and exposure assessment. Five studies were rated as moderate quality (score of 7), reflecting generally acceptable methodologies with minor limitations in specific domains (38, 58–60, 62). The remaining 8 studies were rated as low quality (51, 52, 54, 55, 57, 63–65), with scores ≤6, indicating methodological limitations that may compromise the internal validity of their findings. A detailed summary of the quality assessment is provided in Supplementary Table S3.

#### 3.1.3 Heterogeneity

Several case-control studies were excluded from the meta-analysis due to missing quantitative data on LF levels (38, 51, 56) or due to aberrant values that were markedly different from those reported in other studies, which could substantially bias the meta-analysis (59, 62, 63). Attempts to obtain missing data by contacting the corresponding authors were unsuccessful.

For the quantitative synthesis, studies were stratified first by the biological matrix (unstimulated saliva, stimulated saliva, or GCF), and then by periodontitis type. One study included both stimulated and unstimulated saliva and was analysed in both subgroups (57).

##### 3.1.3.1 Unstimulated Saliva

**Figure 2:**
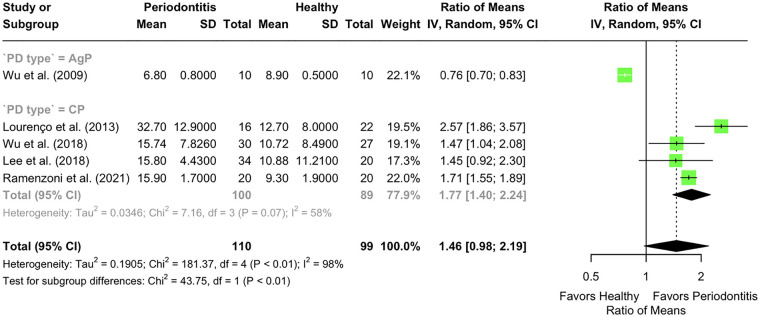
Lactoferrin levels in unstimulated saliva (µg/mL) of individuals with periodontitis vs. those with a healthy periodontium. Subgroup analysis according to the presence of chronic (CP) or aggressive (AgP) periodontitis.

Five studies reported LF concentrations in unstimulated saliva. In the sole AgP study, the ROM was 0.76 (95% CI: 0.70–0.83) (60). In contrast, 4 studies focusing on CP reported a pooled ROM of 1.77 (95% CI: 1.44–2.24; *p* < 0.001) (53, 57, 61, 64). Heterogeneity in this subgroup was moderate (*I*^2^ = 58%, *p* = 0.07). The difference in LF levels between AgP and CP was statistically significant (Chi^2^ = 43.75; df = 1; *p* < 0.01). The overall pooled ROM from all 5 studies was 1.46 (95% CI: 0.98–2.19; *p* < 0.001), with high heterogeneity (*I*^2^ = 98%, *p* < 0.01) (Figure 2).

##### 3.1.3.2 Stimulated Saliva

**Figure 3:**
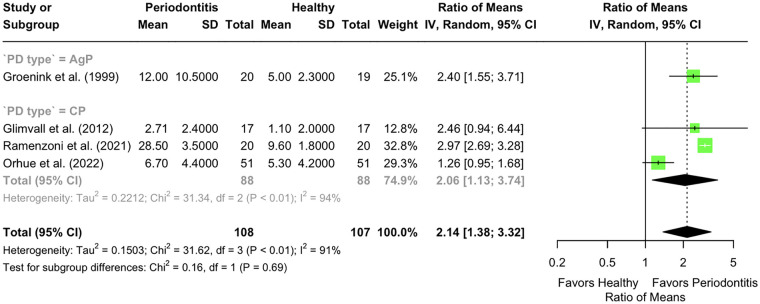
Lactoferrin levels in stimulated saliva (µg/mL) of individuals with periodontitis vs. those with a healthy periodontium. Subgroup analysis according to the presence of chronic (CP) or aggressive (AgP) periodontitis.

Four studies analysed LF in stimulated saliva. One AgP study showed a ROM of 2.40 (95% CI: 1.55–3.71) (53). Three CP studies (55, 57, 65) including 88 patients and 88 controls yielded a pooled ROM of 2.14 (95% CI: 1.38–3.32; *p* < 0.001), with high heterogeneity (*I* ^2^= 94%, *p* < 0.01). The difference in LF levels between AgP and CP subgroups was not statistically significant (Chi^2^ = 0.16; df = 1; *p* = 0.69). Combining both periodontitis types, the overall ROM was 2.14 (95% CI: 1.38–3.32; *p* < 0.001), with substantial heterogeneity (*I*^2^ = 91%, *p* < 0.01) (Figure 3).

##### 3.1.3.3 Gingival crevicular fluid

**Figure 4:**
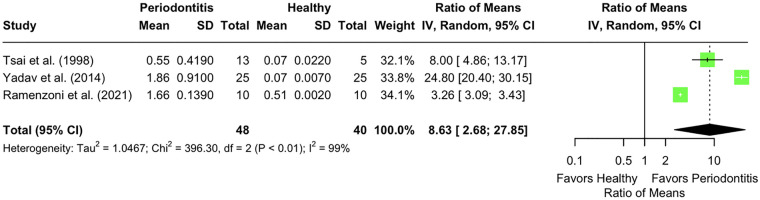
Lactoferrin levels in gingival crevicular fluid (µg/mL) of individuals with chronic periodontitis (CP) vs. those with a healthy periodontium.

Three studies were included that reported LF levels in the GCF of individuals with CP (52, 56, 58). When analysed collectively, these studies yielded a pooled ROM of 8.63 (95% CI: 2.68–27.87; *p* < 0.001) (Figure 4). However, the level of heterogeneity was extremely high (*I*^2^ = 99%; *p* < 0.01), indicating substantial inconsistency in study design, sampling procedures, or analytical methods.

Taken together, across all biological matrices analysed, a consistent pattern was observed whereby LF levels were higher in patients with periodontal disease compared to periodontally healthy controls. This trend was particularly evident in stimulated saliva and GCF, where the magnitude of the ROM indicated substantially elevated concentrations in affected individuals. Despite the presence of heterogeneity among studies, the direction of the effect remained consistent, supporting the association between increased LF levels and periodontal disease status.

### 3.2 Interventional studies

LF has also been investigated as a potential biomarker for monitoring periodontitis by quantifying its levels in unstimulated saliva, stimulated saliva, and GCF before and after mechanical periodontal therapy.

#### 3.2.1 Study characteristics

This subanalysis of a systematic review encompassed 6 interventional studies conducted in India (52, 66), Taiwan (53), Germany (67), Finland (51), and Ghana (93). While not all studies provided sex-specific data, the reported information indicates a balanced male-to-female ratio of 0.96. Precise mean age estimation was not possible due to most studies reporting only age ranges without detailed distributions.

All studies excluded smokers, except for Jentsch et al. (67), who included participants with smoking habits, systemic diseases, and/or chronic medication use. Regarding periodontitis types, Suomalainen et al. (51) included patients with LJP, whereas the others focused on CP. Sample types varied: 3 studies used unstimulated saliva, 2 used stimulated saliva, and 4 analysed GCF. For LF quantification, all studies employed ELISA, except Suomalainen et al. (51), who used an IEMA.

#### 3.2.3 Lactoferrin levels before and after periodontal therapy

In unstimulated saliva, a significant post-therapeutic reduction in LF levels was observed, particularly among patients with CP. Jentsch et al. (67) reported a statistically significant decline in baseline LF concentrations from 9.00 ± 4.19 µg/mL to 7.11 ± 3.12 µg/mL at 14 days following mechanical periodontal therapy (*p* = 0.007). Similarly, Lee et al. (53) documented a significant reduction in salivary LF levels 4 to 6 weeks after treatment, with median concentrations decreasing from 15.801 µg/mL (IQR: 12.707–18.687) to 12.208 µg/mL (IQR: 5.586–17.090) (*p* = 0.020). However, Ayettey-Adamafio et al. (93) found no statistically significant changes in salivary LF levels four weeks after non-surgical periodontal treatment in patients with AgP, despite slight variations in generalized aggressive periodontitis (GAP) subgroup (Table 4).

**Table 4:** Main characteristics and findings of published studies on lactoferrin levels in unstimulated saliva, stimulated saliva, and gingival crevicular fluid of individuals with periodontal diseases, before and after periodontal treatment.

Author and year	Country	Periodontal diagnosis N (male/female)	Age mean ± SD (range)	Exclusion criteria	Sample	Pre-tratment and post-treatment lf levels (time interval)	*p*-value
Suomalainen et al. (51)	Finland	LJP: 7 (1m/6f)	LJP: 17.5y(14–20y)	Smokers	Stimulated saliva	NA	NS
Suomalainen et al. (51)	Finland	LJP: 7 (1m/6f)	LJP: 17.5y(14–20y)	Smokers	GCF	NA	NS
Jentsch et al. (67)	Germany	CP: 25 (12m/13f)	CP: 40.8 ± 9.6y(NA)	(a)	Unstimulated saliva	PRE-: 9.00 ± 4.19 µg/mL POST-:7.11 ± 3.12 µg/mL^†^ (2 wk)	0.007
					Stimulated saliva	PRE-: 10.54 ± 4.58 µg/mL POST-: 8.96 ± 4.22 µg/mL^†^ (2 wk)	0.004
					GCF	PRE-: 1.63 ± 0.53 µg/mL POST-: 1.23 ± 0.59 µg/mL^†^ (2 wk)	0.004
Yadav et al. (52)	India	GCP: 25 (NA)	Total: NA(25–54y)	Smokers; Systemic diseases; Pregnancy/Nursing	GCF	PRE-: 1.86 ± 0.09 µg/mL POST-: 1.41 ± 0.08 µg/mL^†^ (4 wk)	0.003
Kivadasannavar et al. (66)	India	CP: 30 (18m/12f)	CP: 40.5y(28–52y)	Smokers; Systemic diseases; Pregnancy; Hormonal contraceptives; Antibiotic consumption	GCF	PRE-: 266.53 ± 75.86U/mL POST-: 195.47 ± 74.53 U/mL^†^ (2 w) POST-: 90.42 ± 32.89 U/mL^‡^ (2 wk)	<0.0001
Lee et al. (58)	Taiwan	CP: 34 (16m/18f)	CP: NA (23–78y)	Smokers; Systemic diseases	Unstimulated saliva	PRE-: 16.91 ± 4.43 µg/mL^§^ POST-: 15.00 ± 11.21 µg/mL^†^^,^^§^ (4 wk)	0.020
Ayettey-Adamafio et al. (91)	Ghana	LAP: 9 (6m/3f) GAP: 10 (4m/6f)	LAP: 32.11 ± 8.07y GAP: 33.80 ± 8.93y (20–50y)	Comorbid conditions; Use of antibiotics or medications for systemic diseases; Known alcoholics; Smokers; Pregnant or lactating women; Had received SRP in the previous six months	Unstimulated saliva	PRE-(LAP): 70.08 ± 26.12 µg/mL POST-(LAP): 74.40 ± 15.42 µg/mL^†^ (4 wk) PRE-(GAP): 76.94 ± 20.12 µg/mL POST-(GAP): 63.84 ± 31.81 µg/mL^†^ (4 wk)	NS

N, number of participants; SD, standard deviation; m/f, male/female; LJP, localized juvenile periodontitis; CP, chronic periodontitis; GCP, Generalized Chronic periodontitis; LAP, localized aggressive periodontitis; GAP, generalized aggressive periodontitis; NA, not available; y, years; (a), Smokers, systemic diseases, and medication were included; GCF, gingival crevicular fluid; LF, lactoferrin; SRP, scaling and root planning; PRE-, pre-treatment; POST-,Post-treatment; wk, weeks; m, months; NS, not significant.

† Scaling and root planning.

‡ Periodontal surgery.

§ Calculated based on the median and interquartile range.

In contrast, LF changes in stimulated saliva were less consistent. Among patients with LJP, Suomalainen et al. (51) reported a non-significant reduction in LF concentration post-treatment; however, specific values were not disclosed. In CP patients, Jentsch et al. (67) observed a significant decrease in LF levels from 10.54 ± 4.58 µg/mL to 8.96 ± 4.22 µg/mL (*p* = 0.004).

Regarding GCF, no statistically significant variation in LF levels was found in LJP patients following treatment (51). Conversely, among individuals with CP, Jentsch et al. (67) reported a significant reduction in LF concentrations at 14 days post-treatment, from 1.63 ± 0.53 µg/mL to 1.23 ± 0.59 µg/mL (*p* = 0.004). Additionally, Kivadasannavar et al. (66) noted a marked decrease in LF levels from 266.53 ± 75.86 U/mL to 195.47 ± 74.53 U/mL after scaling and root planning, with a further significant reduction to 90.42 ± 32.89 U/mL following periodontal surgery (*p* < 0.0001).

#### 3.2.4 Risk of bias and certainty of evidence

The ROBINS-I assessment revealed a high risk of bias due to confounding, participant selection, and outcome measurement in several studies, compromising their internal validity. Only the study by Lee et al. (53) showed an overall low risk, demonstrating strong methodological quality (Supplementary Table S4).

For case-control studies, the certainty of evidence was rated as low for all outcomes (unstimulated saliva, stimulated saliva, and GCF), due to risk of bias and high heterogeneity. The ROM ranged from 1.77 to 8.63, indicating significantly higher LF concentrations in periodontitis patients compared to healthy controls. For intervention studies, the certainty of evidence was also rated as low, due to risk of bias and indirectness. A reduction in LF levels was observed across different sample types 2–4 weeks after non-surgical periodontal treatment, although the effect size could not be calculated (Supplementary Table S6).

## 4 Discussion

This systematic review analysed 19 studies assessing the relationship between salivary LF levels and periodontal disease, of which 11 met criteria for meta-analysis. Most included studies were conducted in Europe and Asia, primarily using case-control or pre-/post-treatment designs. CP was the most frequently studied clinical condition, and samples included unstimulated saliva, stimulated saliva, and GCF. ELISA was the predominant analytical technique. A key finding of the present meta-analysis is that LF levels are elevated in individuals with periodontal disease across different biological matrices. This pattern remained consistent regardless of the type of saliva analysed (stimulated or unstimulated) or the use of gingival crevicular fluid, suggesting that increased lactoferrin is a robust and reproducible marker of periodontal inflammation. However, high heterogeneity—stemming from variation in study design, population characteristics, and analytical methods—limited the comparability of findings and precluded pooled analysis of intervention outcomes. While reductions in LF levels following therapy were consistently reported, the lack of uniform treatment protocols and timing of post-treatment sampling impaired statistical integration. Notably, this is the first systematic review focused exclusively on LF as a potential biomarker in periodontitis. Previous reviews addressing salivary biomarkers only briefly mentioned LF and included few studies with methodological weaknesses (68, 69).

The main methodological biases identified in the studies included in this review involve participant selection, sample collection and storage, as well as the analytical procedures employed. Below, we discuss the primary sources of bias reported in the literature, which are detailed in Supplementary Table S5.

Several studies relied on convenience sampling without performing prior sample size calculations, thereby reducing statistical power and increasing the risk of bias (51, 59, 63). Additionally, many lacked detailed demographic reporting, particularly with respect to ethnicity (58), or presented imbalances in sex distribution (51). Age discrepancies between case and control groups were also frequently observed (54), despite the well-documented influence of age and sex on salivary flow rate and protein composition, including LF (70–72).

Moreover, several studies failed to adequately control for key confounding variables such as smoking and systemic conditions like diabetes (62, 65). Smoking has been shown to significantly reduce salivary LF concentrations (73), while diabetes can alter the overall salivary proteome (74). The use of medications known to impair salivary gland function—and thereby reduce flow and protein output—represents an additional, often overlooked confounder (75, 76, 78).

Standardization of sampling protocols was inconsistent. Pre-sampling instructions and timing were often unreported, despite evidence that salivary flow and composition vary diurnally (77, 78). Sample collection methods varied substantially—e.g., Yadav et al. (52) used micropipettes, while others employed Periopaper® strips with non-standardized insertion times (63). Volume quantification tools like the Periotron 6000® were sometimes used without calibration (58), undermining measurement reliability. Although LF stability is maintained at −80 °C (79, 80), some studies used suboptimal storage conditions or failed to report storage time or pre-freezing centrifugation (51, 61).

The timing of post-treatment sampling also varied—e.g., Jentsch et al. (67) and Kivadasannavar et al. (66) collected samples at 14 days post-therapy, which may be insufficient to capture LF normalization. A more appropriate sampling window is suggested to be 6–8 weeks post-treatment (81). Most studies used ELISA for LF quantification, yet calibration details were often omitted [e.g., Friedman et al. (63); Wei et al. (59); Groenink et al. (54)]. This omission can impact assay sensitivity and specificity (56, 82).

Consistently elevated LF levels in both saliva and GCF among CP patients suggest that LF may serve as a useful biomarker for periodontal disease. Given LF's origin from neutrophils and its increase in inflammatory contexts (83, 84) elevated levels—particularly in GCF—likely reflect neutrophil activation. Since neutrophils constitute ∼98% of GCF cellular content (85, 86), LF concentration in this fluid strongly correlates with periodontal pocket depth and disease severity (87, 88).

Evidence also indicates that LF levels decline following periodontal therapy, especially in non-smokers (89). This is reflected in reduced neutrophil recovery in oral rinses (90) and diminished LF concentrations in both saliva and GCF post-treatment (52, 67). These changes reinforce LF's potential as a dynamic marker of inflammation and treatment response.

Genetic variability may also contribute to differences in LF levels and function. Several studies have identified single nucleotide polymorphisms (SNPs) in the LF gene, such as rs1126477 and rs1126478, which are associated with susceptibility to chronic and aggressive periodontitis (92, 93). These variants can alter the antimicrobial activity and immunomodulatory properties of LF, potentially influencing host–microbe interactions and disease progression (94). Moreover, recent evidence suggests that specific polymorphisms, such as rs1126478, may modulate LF levels in GCF and act as predictors of disease phenotype and severity (95).

Clinically, LF could enhance early detection and monitoring of periodontal disease, particularly in settings lacking access to specialized care. However, for LF to transition into routine clinical use, key challenges must be addressed. These include standardizing sampling and analytical methods, controlling for patient-related confounders, and conducting large-scale validation studies in diverse populations (96, 97). Only through such efforts can the reliability and generalizability of salivary LF as a periodontal biomarker be fully established.

From this perspective, LF can be detected in saliva and GCF using several laboratory-based techniques. The most widely used method is ELISA, due to its high sensitivity and specificity, and it has been employed in most periodontal studies (53–57). Alternative methods include immunoenzymometric assays (IEMA) and electroimmunodiffusion, although these have been less frequently used (51, 63).

Importantly, emerging diagnostic platforms are being developed to enable point-of-care detection of salivary biomarkers through rapid immunochromatographic strip tests and biosensor-based technologies (98). Although these approaches remain largely in the experimental or early translational stage and are not yet integrated into routine clinical practice, they represent a transformative direction in periodontal diagnostics. Their capacity to deliver real-time, non-invasive, and potentially cost-effective assessments positions them as promising tools for future chairside applications, with the potential to shift periodontal diagnosis toward earlier detection, improved risk stratification, and more personalised disease monitoring (56, 99).

## 5 Conclusion

Salivary and GCF LF levels are significantly elevated in patients with periodontal disease, particularly in chronic periodontitis, compared to periodontally healthy individuals, while the evidence is less clear for aggressive periodontitis. LF levels decrease substantially following mechanical periodontal therapy. The inherent advantages of LF as a potential biomarker underscore the need for future investigations using more rigorous and standardized protocols to produce robust and reproducible results. Such studies are essential to definitively establish the clinical utility of LF for the diagnosis and monitoring of periodontal disease.

## Data Availability

The original contributions presented in the study are included in the article/Supplementary Material, further inquiries can be directed to the corresponding author.
