# Supplementary material for: The role of salivary lactoferrin as a potential biomarker for periodontal disease: a systematic review and meta-analysis
Source: Front Oral Health. 2026 May 22;7:1812772. doi: 10.3389/froh.2026.1812772 (PMC13236940; doi:10.3389/froh.2026.1812772)
Supplement: Supplementary file 1 [file Table1.docx]

**Table S1.** Search strings used for each database queried.

| **Database** | **Search strings** |
| --- | --- |
| Pubmed | (((lactoferrin) OR (lactotransferrin)) AND ((periodontal disease) OR (periodontitis))) |
| Scopus | (((lactoferrin) OR (lactotransferrin)) AND ((periodontal disease) OR (periodontitis))) |
| Web of science | (((lactoferrin) OR (lactotransferrin)) AND ((periodontal disease) OR (periodontitis))) (All Fields) |
| Embase | (((lactoferrin) OR (lactotransferrin)) AND ((periodontal disease) OR (periodontitis))) (All Fields) |
| Cochrane Library | ("Lactoferrin":ti,ab,kw OR "Lactotransferrin":ti,ab,kw) AND ("Periodontal disease":ti,ab,kw OR "Periodontitis":ti,ab,kw) |
