# Supplementary material for: The role of salivary lactoferrin as a potential biomarker for periodontal disease: a systematic review and meta-analysis
Source: Front Oral Health. 2026 May 22;7:1812772. doi: 10.3389/froh.2026.1812772 (PMC13236940; doi:10.3389/froh.2026.1812772)
Supplement: Supplementary file 2 [file Table2.docx]

**Table S2.  List of excluded articles and reasons for exclusion.**

| **Study** | **Reason for exclusion** |
| --- | --- |
| Velliyagounder, K., Kaplan, J. B., Furgang, D., Legarda, D., Diamond, G., Parkin, R. E., & Fine, D. H. (2003). One of two human lactoferrin variants exhibits increased antibacterial and transcriptional activation activities and is associated with localized juvenile periodontitis. Infection and immunity, 71(11), 6141–6147. https://doi.org/10.1128/IAI.71.11.6141-6147.2003 | Wrong study design |
| Iozon-Ene, S., Soancă, A., Roman, A., Páll, E., Ciurea, A., Oneț, D., Stanomir, A., Popescu, D. M., & Micu, I. C. (2021). The functions of lactoferrin in the oral cavity. Romanian Journal of Stomatology, 67(3), 143–149. https://doi.org/10.37897/RJS.2021.3.2 | Wrong study design |
| Atia, N. S. M., El-Nemr, R. A., & Abo-Elsoud, A. A. E. (2023). Effect of lactoferrin on enamel characteristics of primary and permanent teeth: an in-vitro study. BMC oral health, 23(1), 993. https://doi.org/10.1186/s12903-023-03709-1 | Wrong study design |
| Xu, J., Xu, W., Dong, Y., Deng, Z., Jiang, X., Yuan, Y., Fang, J., Wan, Y., & Ren, Y. (2023). Study on inflammatory immune mechanism of lactoferrin in the treatment of periodontitis. Chinese Journal of Modern Applied Pharmacy, 40(15), 2086–2092. https://doi.org/10.13748/j.cnki.issn1007-7693.20223405 | Wrong study design |
| Janani, M., Maghimaa, M., & Rajamanikam, R. (2025). Therapeutic potential of antimicrobial peptides against dental pathogens: A review. International Journal of Advanced Science and Engineering, 12(2), 5714–5737. https://doi.org/10.29294/IJASE.12.2.2025.5714-5737 | Wrong study design |
| Gustafsson, A., Asman, B., & Bergström, K. (1994). Elastase and lactoferrin in gingival crevicular fluid: possible indicators of a granulocyte-associated specific host response. Journal of periodontal research, 29(4), 276–282. https://doi.org/10.1111/j.1600-0765.1994.tb01222.x | Wrong outcome |
| Adonogianaki, E., Moughal, N. A., & Kinane, D. F. (1993). Lactoferrin in the gingival crevice as a marker of polymorphonuclear leucocytes in periodontal diseases. Journal of clinical periodontology, 20(1), 26–31. https://doi.org/10.1111/j.1600-051x.1993.tb01755.x | Wrong study design |
| Husain, J., Griffith, G. S., Rawlingson, A., Stafford, G., & Douglas, C. W. I. (2019). An investigation into the synergistic relationship between lactoferrin and azithromycin with particular reference to periodontopathic bacteria. Materials Today: Proceedings, 16, 2325–2332. | Wrong study design |
| Miyasaki, K. T., Bodeau, A. L., & Flemmig, T. F. (1991). Differential killing of Actinobacillus actinomycetemcomitans and Capnocytophaga spp. by human neutrophil granule components. *Infection and immunity*, *59*(10), 3760–3767. https://doi.org/10.1128/iai.59.10.3760-3767.1991 | Wrong outcome |
| Rhodes, E. R., Menke, S., Shoemaker, C., Tomaras, A. P., McGillivary, G., & Actis, L. A. (2007). Iron acquisition in the dental pathogen Actinobacillus actinomycetemcomitans: what does it use as a source and how does it get this essential metal?. Biometals : an international journal on the role of metal ions in biology, biochemistry, and medicine, 20(3-4), 365–377. https://doi.org/10.1007/s10534-006-9058-3 | Wrong study design |
| Almståhl, A., Wikström, M., & Groenink, J. (2001). Lactoferrin, amylase and mucin MUC5B and their relation to the oral microflora in hyposalivation of different origins. *Oral microbiology and immunology*, *16*(6), 345–352. https://doi.org/10.1034/j.1399-302x.2001.160605.x | Wrong outcome |
| Antequera, D., Sande, L., Mato, E. G., Romualdi, D., Carrero, L., Municio, C., Diz, P., & Carro, E. (2025). Salivary lactoferrin levels in Down Syndrome: a case-control study. Brain, behavior, & immunity - health, 46, 100999. https://doi.org/10.1016/j.bbih.2025.100999 | Wrong study design |
| Guzman, Y. A., Sakellari, D., Papadimitriou, K., & Floudas, C. A. (2018). High-throughput proteomic analysis of candidate biomarker changes in gingival crevicular fluid after treatment of chronic periodontitis. Journal of periodontal research, 53(5), 853–860. https://doi.org/10.1111/jre.12575 | Wrong intervention |
| Brinkmann, O., Zhang, L., Giannobile, W. V., & Wong, D. T. (2011). Salivary biomarkers for periodontal disease diagnostics. Expert opinion on medical diagnostics, 5(1), 25–35. https://doi.org/10.1517/17530059.2011.542144 | Wrong outcome |
| Karthiga Devi, G., Geetha, R. V., Vishnu Priya, V., & Gayathri, R. (2017). Comparative analysis of salivary protein in individuals with and without periodontitis. International Journal of Pharmaceutical Sciences Review and Research, 43(1), 23–24. | Wrong outcome |
| Berlutti, F., Pilloni, A., Pietropaoli, M., Polimeni, A., & Valenti, P. (2011). Lactoferrin and oral diseases: current status and perspective in periodontitis. Annali di stomatologia, 2(3-4), 10–18. | Wrong study design |
| Ferreira, S. M., Gonçalves, L. S., Torres, S. R., Nogueira, S. A., & Meiller, T. F. (2015). Lactoferrin levels in gingival crevicular fluid and saliva of HIV-infected patients with chronic periodontitis. Journal of investigative and clinical dentistry, 6(1), 16–24. https://doi.org/10.1111/jicd.12017 | Wrong outcome |
