# Supplementary material for: The role of salivary lactoferrin as a potential biomarker for periodontal disease: a systematic review and meta-analysis
Source: Front Oral Health. 2026 May 22;7:1812772. doi: 10.3389/froh.2026.1812772 (PMC13236940; doi:10.3389/froh.2026.1812772)
Supplement: Supplementary file 3 [file Table3.docx]

**Table S3.** Quality assessment for case-control studies (Risk of bias analysis assessed using the Newcastle-Ottawa Scale).

|  | **Selection** | | | | **Comparability** | **Exposure** | | | **Overall** |
| --- | --- | --- | --- | --- | --- | --- | --- | --- | --- |
| **Study** | **Case definition adequate**  **(maximum ●)** | **Representiveness of cases**  **(maximum ●)** | **Selection of controls (maximum ●)** | **Definition of controls (maximum ●)** | **Based on design and analysis**  **(maximum ●●)** | **Ascertainment of exposure**  **(maximum ●)** | **Same method of ascertainment for cases and controls**  **(maximum ●)** | **Non-response rate**  **(maximum ●)** |  |
| Friedman et al., 1983 |  |  |  |  |  | ● | ● |  | 2 |
| Tsai et al., 1998 | ● | ● | ● | ● | ● | ● | ● |  | 7 |
| Suomalainen et al., 1996 | ● | ● | ● |  | ●● |  | ● |  | 6 |
| Groenink et al., 1999 | ● | ● |  | ● | ●● |  | ● |  | 6 |
| Wei et al., 2004 | ● | ● | ● | ● | ● | ● | ● |  | 7 |
| Wu et al., 2009 | ● | ● |  | ● | ●● | ● | ● |  | 7 |
| Glimvall et al., 2012 | ● | ● |  | ● |  | ● | ● |  | 5 |
| Lourenço et al., 2013 | ● |  |  | ● | ● | ● | ● |  | 5 |
| Fine et al., 2013 | ● | ● | ● | ● | ● | ● | ● |  | 7 |
| Yadav et al., 2014 | ● | ● |  |  | ● | ● | ● |  | 5 |
| Wu et al., 2018 | ● | ● | ● | ● | ●● | ● | ● |  | 8 |
| Lee et al., 2018 | ● | ● | ● | ● | ●● | ● | ● | ● | 9 |
| Ramenzoni et al., 2021 (1) | ● | ● | ● | ● | ●● | ● | ● | ● | 9 |
| Ramenzoni et al., 2021 (2) | ● | ● |  |  | ● | ● | ● | ● | 6 |
| Orhue et al., 2022 | ● | ● |  |  | ●● | ● | ● |  | 6 |
| Arab et al., 2024 | ● | ● | ● |  | ● | ● | ● | ● | 7 |

●: 1 point

**Table S4.** Quality assessment of intervention studies using the ROBINS-I tool.

| **Study** | **Risk of bias domains** | | | | | | | |
| --- | --- | --- | --- | --- | --- | --- | --- | --- |
|  | **Bias due to confounding** | **Bias due to selection of participants** | **Bias in classification of interventions** | **Bias due to deviations from intended interventions** | **Bias due to missing data** | **Bias in measurement of outcomes** | **Bias in selection of reported results** | **Overall risk** |
| Suomalainen et al., 1996 |  |  |  |  |  |  |  | Serious |
| Jentsch et al., 2004 |  |  |  |  |  |  |  | Serious |
| Yadav et al., 2014 |  |  |  |  |  |  |  | Serious |
| Kivadasannavar et al., 2014 |  |  |  |  |  |  |  | Serious |
| Lee et al., 2018 |  |  |  |  |  |  |  | Serious |
| Ayettey-Adamafio et al. 2025 |  |  |  |  |  |  |  | Serious |

**Judgement:**

**Low risk**

**Moderate risk**

**Serious risk**

**Critical risk**

**Table S5.** Potential methodological biases identified in published studies on lactoferrin levels and periodontal diseases.

| AUTHOR AND YEAR | PATIENT SELECTION | SAMPLE COLLECTION AND STORAGE | SAMPLE ANALYSIS |
| --- | --- | --- | --- |
| Friedman et al., 1983 | Sample size not calculated  Race/ethnicity unspecified  Differences in case-control age ranges | No prior instructions (participants)  No timetable  Paper strips left in the sulcus for less than 3 minutes  No time specified until processing | Electroimmunodiffusion technique  Reader calibration unspecified  Outlier results |
| Suomalainen et al., 1996 | Sample size not calculated  Predominantly female participants | No prior instructions (participants)  Saliva samples stored at -20ºC  No time specified until processing | IEMA technique  Lactoferrin values for the periodontitis group are not provided in the study |
| Tsai et al., 1998 | Sample size not calculated  Race/ethnicity unspecified  Differences in case-control age ranges | Sample collection (GCF) for 30 seconds  Periotron 6000® calibration unspecified  No time specified until processing | ELISA technique  Absorbance calibration unspecified |
| Groenink et al., 1999 | Sample size not calculated  Differences in case-control age ranges  No exclusion of smokers or systemic diseases/drugs | Saliva samples stored at -20ºC  No time specified until processing | ELISA technique  Absorbance calibration unspecified |
| Jentsch et al., 2004 | No exclusion of smokers or systemic diseases/drugs | Saliva samples stored at -18ºC  No time specified until processing | ELISA technique  Absorbance calibration unspecified |
| Wei et al., 2004 | Sample size not calculated | Sample collection (GCF) for 30 seconds  Saliva samples stored at -20ºC  No time specified until processing | ELISA technique  Absorbance calibration unspecified  Outlier results |
| Wu et al., 2009 | Sample size not calculated | Fasting prior to sample collection  No time specified until processing | ELISA technique  Absorbance calibration unspecified |
| Glimvall et al., 2012 | Sample size not calculated  No exclusion of smokers or diabetics | No time specified until processing | ELISA technique |
| Lourenço et al., 2013 | Sample size not calculated  No exclusion of smokers or diabetics | Rinse with 10 ml of phosphate-buffered saline prior to sample collection  No time specified until processing | ELISA technique  Absorbance calibration unspecified |
| Fine et al., 2013 | Sample size not calculated | No time specified until processing | ELISA technique  Lactoferrin values for the periodontitis group are not provided in the study |
| Yadav et al., 2014 | Sample size not calculated  Very broad age range | Sample collection (GCF) using a microcapillary pipette for 5-20 minutes  Post-treatment sampling only if a good response was observed  No time specified until processing | ELISA technique |
| Kivadasannavar et al., 2014 | Sample size not calculated | Sample collection (GCF) using a microcapillary pipette (collection time unspecified) | ELISA technique  Lactoferrin values are in non-convertible units |
| Wu et al., 2018 | Sample size not calculated | Fasting prior to sample collection  Saliva samples stored at -20ºC for 6 months | ELISA technique |
| Lee et al., 2018 | Sample size not calculated | Sample collection during two time slots  Saliva samples stored at -20ºC for 6 months | ELISA technique |
| Ramenzoni et al., 2021 (1) | Sample size not calculated  Very strict exclusion criteria | Sample collection (GCF) for 30 seconds  Saliva samples stored at -20ºC  GCF samples stored at -80ºC for 6 months | ELISA technique  Lactoferrin values for the periodontitis group are not provided in the study |
| Ramenzoni et al., 2021(2) | Sample size not calculated  No exclusion of smokers | Time until processing not specified | ELISA technique |
| Orhue et al., 2022 | Sample size not calculated  Does not use the 2018 periodontitis classification | Rinse with water for 30 seconds before sampling  No time specified until processing | ELISA technique  Absorbance calibration unspecified |
| Arab et al., 2024 | Very broad age range (20-89 years)  Includes individuals with cardiovascular disease, diabetes, and smokers  Does not use the 2018 periodontitis classification | No prior instructions (participants)  No time specified until processing | ELISA technique  Outlier results |
| Ayettey-Adamafio et al. 2025 | Does not use the 2018 periodontitis classification | Time until processing not specified | ELISA technique  Absorbance calibration unspecified |

GCF: Gingival Crevicular Fluid; IEMA: immunoenzymometric assay; ELISA: Enzyme-Linked Immunosorbent Assay
